# Knockdown of Plexin C1 induces epithelial-to-mesenchymal transition and confers resistance to multikinase inhibitors in hepatocellular carcinoma cells

**DOI:** 10.55730/1300-0152.2739

**Published:** 2025-01-08

**Authors:** Gamze GÜNGÖR TOPCU, Arzu AYSAN, Şevval DİK, Maide ŞEKER, Melike Binnur BAHÇEKAPILI, Sude TOPKARAOĞLU, Dilek YAVUZER, Tamer YAĞCI

**Affiliations:** 1 Department of Molecular Biology and Genetics, Faculty of Sciences, Gebze Technical University, Kocaeli, Turkiye; 2 Central Research Laboratory (GTU-MAR), Gebze Technical University, Kocaeli, Turkiye; 3 Department of Histology and Embryology, Hamidiye Faculty of Medicine, University of Health Sciences, İstanbul, Turkiye; 4 Department of Pathology, Hamidiye Faculty of Medicine, University of Health Sciences, Sancaktepe Şehit Prof. Dr. Ilhan Varank Training and Research Hospital, İstanbul, Turkiye

**Keywords:** Plexin C1, hepatocellular carcinoma, epithelial-to-mesenchymal transition, multikinase inhibitors

## Abstract

**Background/aim:**

HCC is a common and lethal malignancy and multi-kinase inhibitors (MKIs) are among the therapeutic options for unresectable cases. However, response rates to MKIs remained variable, necessitating the identification of predictive biomarkers. Plexin C1 (PLXNC1), a receptor involved in cell signaling, has emerged as a potential candidate to regulate tumor responses. This study aims to evaluate the impact of PLXNC1 expression on the sensitivity of HCC cells to MKI therapy.

**Materials and methods:**

shRNA-mediated PLXNC1 knock-down and control clones of HCC cell lines PLC/PRF/5 and Hep3B were generated, and downregulation of PLXNC1 was confirmed using Western blotting. The effects of MKIs sorafenib and lenvatinib on apoptotic cell death and proliferation of HCC cell clones were explored in relation to PLXNC1 expression. Furthermore, tumor responses to MKIs were evaluated in mouse xenograft models engrafted with shPLXNC1 and control clones of PLC/PRF/5 cells.

**Results:**

The results of our in vitro studies indicate that PLXNC1 expression is linked to heightened sensitivity of HCC cells to MKIs. Furthermore, the knockdown of PLXNC1 in these cells resulted in a reduction in proliferation and an increase in apoptosis resistance. The findings were validated in hepatocellular carcinoma (HCC) tumor models in immunodeficient mice, which revealed that cells expressing PLXNC1 were responsive to drug treatment. In PLXNC1-silenced cells, tumor volumes remained stationary, which was attributable to the antiproliferative effect of PLXNC1 knockdown.

**Conclusion:**

PLXNC1 expression may serve as a predictive biomarker for MKI efficacy in HCC and provides a potential avenue for personalized therapeutic strategies. Further clinical validation is required to incorporate PLXNC1 into routine diagnostic and treatment protocols for HCC.

## 1. Introduction

Liver cancer ranks as the 6th most common primary cancer yet is the 3rd leading cause of cancer-related deaths globally (Sung et al., 2021). Hepatocellular carcinoma (HCC) constitutes 80%–90% of primary liver cancers (Ringelhan et al., 2018). Major causes of HCC include hepatitis B and C viruses, excessive alcohol consumption, diabetes, exposure to toxins like aflatoxins, and nonalcoholic fatty liver disease (Kulik and El-Serag, 2019). The development of HCC is driven by overexpression or dysregulation of growth factors and their receptors such as EGFR, IGFR, VEGFR, HGFR (c-MET), PDGFR, and FGFR, leading to alterations in critical signaling pathways such as Ras/Raf/MEK/ERK, JAK/STAT, PI3K/AKT/mTOR, TGF-β, NF-κB and Wnt/β-catenin (Höpfner et al., 2006; Tripathy et al., 2018).

Treatment strategies for HCC include surgical options like partial hepatectomy and liver transplantation, which are effective in early or intermediate stages. HCC is staged into five categories by the Barcelona Clinic Liver Cancer (BCLC) system. Treatment options for stages 0-B include tumor resection, liver transplantation, trans-arterial chemo- and radioembolization, and radiofrequency ablation, achieving 5-year survival rates up to 70% (Llovet et al., 1999; Llovet et al., 2005). However, most HCC cases are diagnosed at advanced stages (stage C), limiting treatment options and resulting in poor prognosis with an average survival of 8 months (Llovet et al., 2021). Drug treatments for unresectable cases involve small molecule inhibitors and antibodies targeting signaling pathways. Four multi-kinase inhibitors (MKIs) are FDA-approved for HCC treatment: Sorafenib, Lenvatinib, Regorafenib, and Cabozantinib, each targeting various receptor tyrosine kinases. Sorafenib, approved in 2007, extends survival by 3 months by inhibiting c-Raf, B-Raf, MEK, ERK, VEGFR-2/3, PDGFR-β, KIT, and Flt3 (Adnane et al., 2006; Kane et al., 2009). Lenvatinib, approved in 2018, targets VEGFR1/2/3, PDGFR-α, FGFR1/2/3/4, KIT, and RET, providing an additional 1.5 months of survival (Ikeda et al., 2016; Kudo et al., 2018). Resistance to these MKIs often necessitates second-line treatment with Regorafenib and Cabozantinib, which target additional pathways through TIE2, MET and AXL, and provide further clinical benefit (Bruix et al., 2017; Abou-Alfa et al., 2018). The complex nature of HCC and its tendency to develop drug resistance underscore the need for personalized treatment plans and preclinical models that preserve in vivo tumor characteristics. Epithelial-Mesenchymal Transition (EMT) is a crucial process in cancer biology, contributing to tumor heterogeneity, drug resistance, metastasis, and cancer stem cell differentiation. EMT involves the loss of epithelial characteristics and the gain of mesenchymal traits, regulated by transcription factors like Snail, Slug, Twist, ZEB1, and ZEB2, and signaling pathways such as TGF-β, Wnt, Notch, and Hedgehog (Gonzalez and Medici, 2014; Liu et al., 2014). EMT processes in HCC are associated with increased metastatic potential, invasiveness, and resistance to therapies, making EMT a critical target for improving patient outcomes (van Zijl et al., 2009).

Plexin C1, a receptor involved in cell signaling, has been studied for various cancers. In melanoma, the loss of Plexin C1 in metastatic cells correlates with increased metastatic capacity (Lazova et al., 2009; Scott et al., 2009). In nonsmall cell lung cancer (NSCLC), higher Plexin C1 expression compared to healthy lung epithelial cells has been observed, and the interaction of Plexin C1 with its ligand SEMA7A promotes cell migration (Zhang et al., 2017). In gastric cancer, Plexin C1 overexpression increases cell proliferation and migration, while its knockdown reduces these traits and leads to smaller tumors in nude mice (Chen et al., 2020). Our previous studies have shown an inverse relationship between Plexin C1 expression and tumor grade in HCC, with poorly differentiated, advanced tumors expressing lower levels of Plexin C1 (Odabas et al., 2018; Turhal et al., 2022). In addition, we demonstrated that PLXNC1 knockdown induces the expression of EMT markers. It has been well-documented that the activation of the EMT program can lead to chemoresistance in tumor cells (Steinbichler et al., 2018). The present study therefore investigated the responses of HCC cells to sorafenib and lenvatinib treatment on the Plexin C1 axis and explored the potential of Plexin C1 as a molecular marker for assessing the sensitivity of HCC to MKI treatment.

## 2. Materials and methods

### 2.1. Cell culture

Hepatocellular carcinoma cell lines PLC/PRF/5 and Hep3B were purchased from the American Type Culture Collection (ATCC), routinely screened for mycoplasma contamination, and authenticity validation was performed annually by STR analysis. Cells were cultured in high glucose DMEM medium supplemented with 10% FBS (Fetal bovine serum), 1% NEAA (nonessential amino acid), 1% Penicillin/Streptomycin in a humidified air incubator at 37 °C with 5% CO_2_ and were split twice weekly.

### 2.2. Production of lentiviral transduction particles and generation of cell clones with suppressed PLXNC1 expression

Lentiviral particles were generated by cotransfecting PLXNC1 shRNA or control pLKO.1 plasmids with packaging plasmids pCMV-dR8.2 dvpr and pCMV-VSV-G into HEK293T cells using Poly(ethyleneimine) (PEI). Plasmid DNA and PEI were mixed in Opti-MEM at a 1:3 ratio, incubated for 20 min, and added dropwise to the cells. After 36 h, lentiviral particles were collected, filtered (0.45 μm), and stored at −80 °C. PLC/PRF/5 cells were transduced with viral particles in the presence of 8 μg/ml Polybrene and incubated for 48 h. Positive selection was performed using puromycin (4 μg/ml), with the continued selection at 2.5 μg/mL for three passages to eliminate untransfected cells. Western blot analysis confirmed reduced Plexin C1 expression in shPLXNC1 clones and preserved expression in pLKO controls.

### 2.3. Cell proliferation analysis

pLKO and shPLXNC1 cell clones of PLC/PRF/5 and Hep3B cell lines were counted and seeded in E-Plate 96 (Agilent Technologies, USA) at a density of 5 × 10^3^ cells per well in a volume of 100 μL. Then, the E-plates were inserted into the xCELLigence RTCA DP instrument (ACEA Biosciences) within an incubator set at 37 °C and 5% CO_2_. The impedance-based cell index values of the wells, which are indicative of cell number, were documented at 30-min intervals up to 72 min. PLXNC1-dependent proliferation of cells was measured with cell index values being normalized against the initial values recorded at the time of seeding.

### 2.4. Cell viability assay

PLC/PRF/5 and Hep3B cells were exposed to increasing concentrations of sorafenib and lenvatinib, and cell viability was evaluated using the MTS assay. In summary, cells were seeded in 96-well plates and permitted to adhere overnight. Following a series of concentrations of sorafenib and lenvatinib, the MTS (CellTiter 96 AQUeous, Promega) reagent was added to each well in strict accordance with the manufacturer’s instructions. After incubation for 4 h, the absorbance was measured at 490 nm using a microplate reader. The cell viability was then calculated as a percentage relative to the untreated control group. Half-maximal inhibitory concentration (IC50) values were determined from dose-response curves using GraphPad Prism software. IC50 values, representing the drug concentrations required to inhibit 50% of cell viability, provided a quantitative measure of the cytotoxic effects of sorafenib and lenvatinib on cells.

### 2.5. Cell cycle analysis

The effect of PLXNC1 on cell cycle was determined by flow cytometry using PI (Propidium Iodide) staining. Cells were seeded in 6-well plates at a density of 2(×)10^5^ per well. After 48 h, cells were harvested and washed with cold PBS. Then, cells were fixed with 1 mL of 70% cold ethanol for 2 h. Before analysis, cells were centrifuged at 400 g for 5 min and cell pellets were incubated in a solution containing 0.1% TritonX-100, 3 μL RNase A, and 5 μL PI for 90 min at 37 °C. Distribution of cellular DNA content through cell cycle phases was quantified by BD Accuri C6 flow cytometry.

### 2.6. Invasion and migration assay

The Transwell migration assay was performed to evaluate the migratory capacity of pLKO and shPLXNC1 cell clones. Transwell inserts with an 8 μm pore-size polycarbonate membrane (CytoSelect, Cellbiolabs, USA) were presoaked in medium and placed into 24-well plates containing 10% FBS-supplemented medium. Cells (2.5 × 10^5^) were seeded into the inserts and allowed to adhere for 2 h before replacing the medium with serum-free medium. After 48 h, migrated cells on the bottom membrane were fixed, stained with DAPI, and visualized using a Zeiss LSM880 Airyscan confocal microscope. Cell counts were performed using ImageJ software. The Transwell invasion assay was performed by using matrigel-coated inserts (Corning BioCoat, 8 μm pore size) and the protocol described above for the invasion assay was followed. Invading cells were visualized using a Zeiss LSM880 Airyscan confocal microscope and counted with ImageJ software.

### 2.7. Apoptotic cell death analysis

PLC/PRF/5 and Hep3B cell clones were treated for 72 h with the IC50 values of Sorafenib and Lenvatinib, and the apoptosis response of the cells was assessed via PARP cleavage by Western blot analysis. Briefly, 2.5(×)10^5^ cells were seeded in 60 mm cell culture plates and the next day, drugs were applied for 72 h. Then, cells were harvested, and protein lysate was prepared by using SDS lysis buffer (0.5 M Tris pH: 6.8, 4% Glycerol, 1% SDS). Total (p116) and cleaved (p89) PARP were detected by PARP antibody. Drug-induced cellular apoptosis was also evaluated using AnnexinV-7AAD and TMRE staining. Drug-treated cells were then collected for apoptosis analysis and stained with FITC-Annexin V and PI (Invitrogen apoptosis kit) for 60 min in the dark. They were also incubated with 1 μM TMRE for 30 min to see the changes in membrane potential in the cells and all samples were analyzed on a BD Accuri C6 flow cytometry device.

### 2.8. Western blotting

Cells were lysed in 2X SDS lysis buffer (10% Glycerol, 2% SDS in 62.5 mM Tris-HCl) with protease inhibitor cocktail (Roche) and the protein concentrations were quantified by Qubit 3 Fluorometer (Thermo Fisher Scientific). Total protein samples (50 μg) were run on 10% SDS-PAGE and transferred to PVDF membranes (Millipore). After blocking with nonfat dry milk in TBST (50 mM Tris-HCl, pH 7.5. 150 mM NaCl, 0.05% Tween 20), membranes were probed first with primary antibodies at 4 °C overnight and with the secondary antibodies for 1 h at room temperature. Washing steps were performed in TBS-T buffer and after the final wash, membranes were covered with chemiluminescent WesternBright Sirius HRP Substrate (Advansta) and the target proteins were visualized using the ChemiDoc XRS system (Bio-Rad). The following antibodies were used: anti-Plexin C1, clone PE4, homemade monoclonal antibody (Odabas et al., 2018), E-Cadherin (BD Biosciences, 1/3000), Vimentin (Cell Signaling, 1/2000), PARP (Cell Signaling, 1/1000), antimouse-HRP and antirabbit-HRP secondary antibodies (Advansta, 1/5000).

### 2.9. Xenograft modelling

Animals were treated in accordance with the guidelines of the local ethics committee of TÜBİTAK MAM, approved on 01.03.2019 with reference number 16563500-11-20. For the tumor model, PLC/PRF/5 pLKO and PLC/PRF/5 shPLXNC1 cell clones were implanted subcutaneously (SC) in the flank of 6–8 weeks old female BALB/c Nude mice, and when the tumor volume reached 200–250 mm^3^, Sorafenib (30 mg/kg) and Lenvatinib (10 mg/kg) (Kimura et al., 2018; Zhang et al., 2018) were administered to the mice via oral gavage every 3 days for a total of 28 days. Tumor volumes were measured every 2–3 days with a digital caliper and calculated using the formula L (×)W^2^/2 (L: tumor length, W: tumor width) (Jung, 2014), and monitored Pearl Trilogy imaging system. Tumor tissues removed on the 28th day were photographed and preserved in 10% neutral formalin for IHC analysis.

### 2.10. Immunohistochemistry staining

Immunohistochemistry was performed as previously described (Odabas et al., 2018). Briefly, after deparaffinization and antigen retrieval processes, tissue sections were stained with anti-Plexin C1 and anti-Ki67 antibodies (MIB-1, Dako), and processed in the BenchMark-XT Autostainer (Ventana Medical Technologies, Roche, Germany) device. OptiView DAB IHC detection kit (Roche) was used to visualize the stained tissue and and the tissue sections were analyzed under a Nikon light microscope at 400X magnification. The antibody staining scores (immune reactive score, IRS) of the tissues were created by scoring the tumor staining percentage and tumor staining intensities and multiplying them. Tumor staining percentage was scored as follows: <10% = 0, 10%–25% = 1, 26%–50% = 2, 51%–75% = 3, >75% = 4. Tumor staining intensity was scored as 0 (negative); 1 (weak staining); 2 (moderate staining); and 3 (strong staining). The final percent staining values were calculated according to the following formula: Percent staining: (Sample IRS/Maximum IRS) (×) 100; with the maximum IRS set at 12.

### 2.11. TUNEL analysis

TdT-dUTP nick-end-labelling (TUNEL) assay was performed by using the One-step TUNEL In Situ Apoptosis Kit (Red, Elab Fluor 594) and according to manufacturer’s instructions. Briefly, tissue sections were transferred onto positively charged slides, and after overnight deparaffinization in a 60 °C oven and rehydration in a series of graded alcohols, slides were treated with Proteinase K (20 μg/mL) for 15 min at room temperature. Then, tissues were incubated at 37 °C with 100 μL TdT Equilibration Buffer for 20 min and with 50 μL labeling solution for 60 min. Nuclei were stained with DAPI. PBS was used as a washing reagent between steps. For positive control and to generate DNA ends for the end-labeling reaction, Proteinase K-treated section was incubated with 1 μg/mL of DNase I for 60 min at 37 °C and the reaction was terminated by washing samples with PBS for 15 min. Slides were sealed with coverslips and samples were observed using a fluorescent microscope (Zeiss, Axio Cam) with a digital camera attachment. The emission value of the fluorescence microscope was set to 594 nm and all cells reflecting red light were considered TUNEL (+) apoptotic cells. Three images per sample were recorded at 200X magnification and their analyses were performed blindly (Wu et al., 2017). The following formula was used to determine the apoptotic index (AI) of tissue sections: AI (%) = [TUNEL (+) cell number (×) 100]/Total Cell Number (Malkoç et al., 2020).

### 2.12. Statistical analysis

The density of Western blot bands and nucleus counts in invasion/migration experiments were analyzed using the ImageJ program. Statistical analyses were performed using the GraphPad Prism 8 program using “Student’s t-test”.

## 3. Results

### 3.1. Knockdown of PLXNC1 Confers EMT characteristics on HCC cells

**Figure 1:**
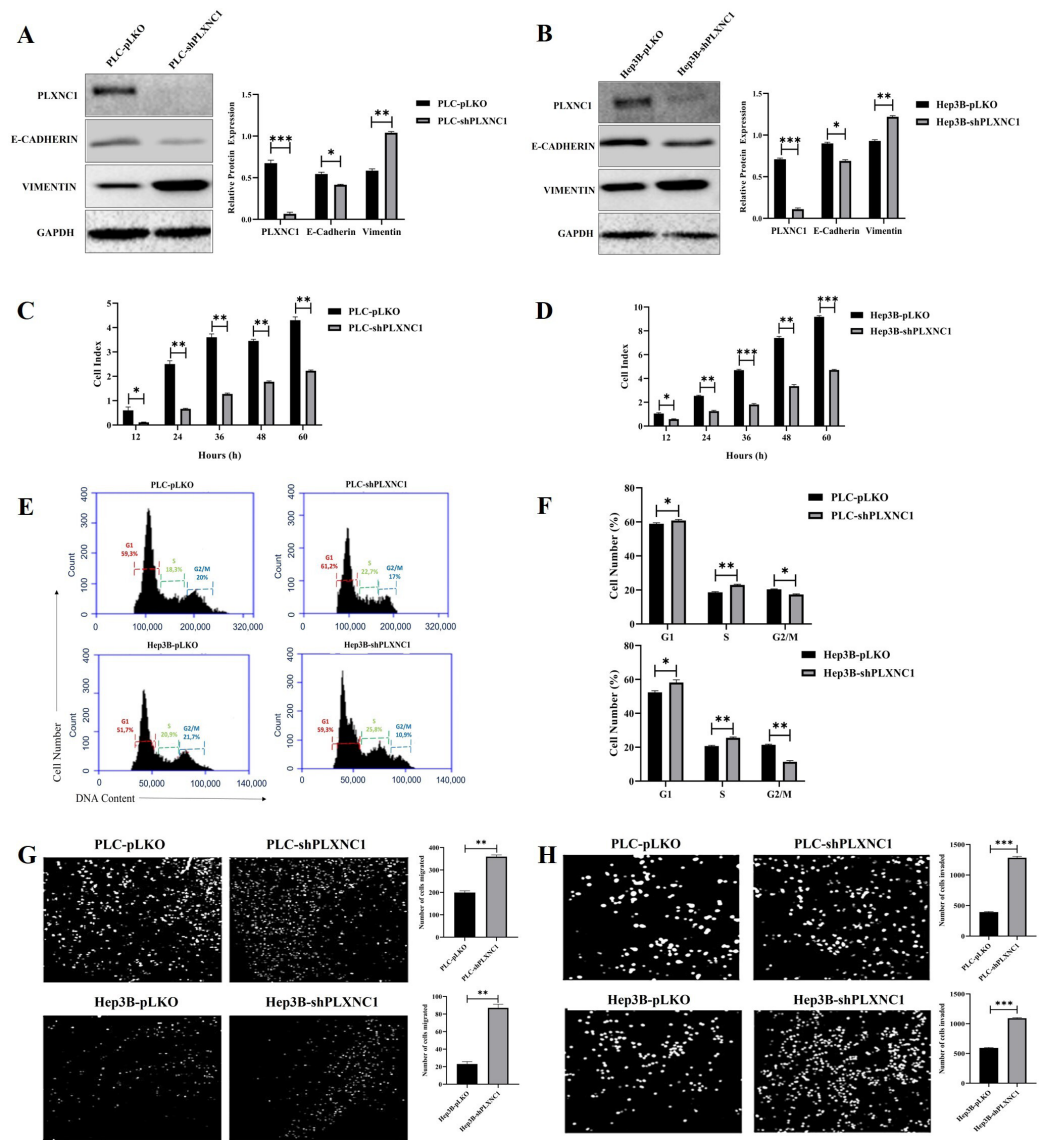
shPLXNC1 clones of HCC cells show increased migration, invasion and expression of EMT markers, but reduced proliferation compared to PLXNC1 expressing PLKO clones. Western blot images show PLXNC1, E-CADHERIN and VIMENTIN expression in shPLXNC1 and pLKO control clones of PLC/PRF/5 (A) and Hep3B cell lines (B). Bar graphics represent the relative protein expression as calculated by the ratio of the intensities of samples to loading control GAPDH. The proliferation rate of the cell clones was monitored real-time for 60 h and represented as cell index for shPLXNC1 and control clones of PLC/PRF/5 (C) and Hep3B cells (D). Flow cytometry histograms show cell cycle distribution of pLKO and shPLXNC1 clones of PLC/PRF/5 and Hep3B cells (E). Bar charts compare differential distribution of pLKO and shPLXNC1 cells in G1, S and G2/M phases of the cell cycle (F). Representative images of trans-well migration and invasion of pLKO and shPLXNC1 cells are shown in (G) and (H), respectively. The number of cells undergoing migration and invasion is quantified by counting the cells on ImageJ program, and bar diagrams representing the observed cell distribution on trans-well membranes are generated (G–H). The statistical analyses are conducted using Student’s t-test on the GraphPad Prism 8 software (*p < 0.05; **p < 0.01; ***p < 0.001).

First, we assessed the efficiency of PLXNC1 knockdown and induction of EMT in shPLXNC1 and PLKO control clones of PLC and Hep3B cells by a WB analysis. The protein expression of PLXNC1 was substantially decreased accompanied by the downregulation of E-Cadherin and upregulation of a mesenchymal marker Vimentin in shPLXNC1 clones of HCC cells (Figures 1A–1B). Cell clones were also subjected to a real-time proliferation analysis for 60 h, and shPLXNC1 cells displayed slower proliferation compared to control clones (Figures 1C–1D). A marked decrease of proliferation was detected in both shPLXNC1-PLC and shPLXNC1-Hep3B cells at all-time points. These results were in accordance with the data obtained in cell cycle analysis. shPLXNC1 cells accumulated at G1 and S phases and their levels significantly decreased at G2 phase; however, the variations were more apparent in shPLXNC1-Hep3B cells compared to shPLXNC1-PLC (Figures 1E–1F). Activation of the EMT program in shPLXNC1 clones was consistent with increased migration and invasion of the cells. Migration and invasion capacities of both shPLXNC1 knockdown clones of Hep3B and PLC cells were significantly increased compared to control clones (Figures 1G–1H).

### 3.2. shPLXNC1 clones of HCC cells resist the effects of multikinase inhibitor treatment

**Figure 2:**
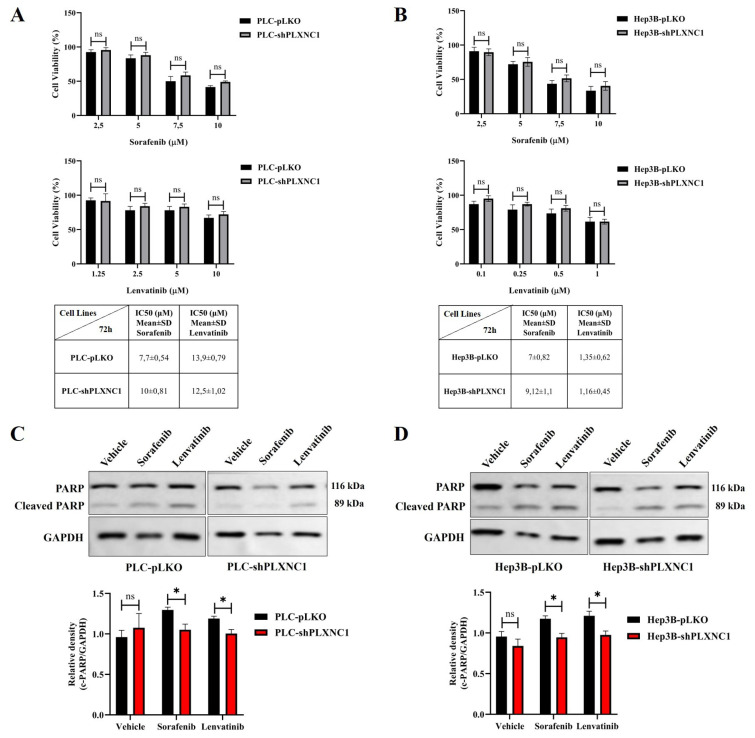
The impact of sorafenib and lenvatinib treatment on cell viability and the cleavage of PARP in pLKO and shPLXNC1 clones of PLC and Hep3B cells. Cell viability of sorafenib- and lenvatinib-treated pLKO and shPLXNC1 clones of PLC and Hep3B are shown in (A) and (B), respectively. Western blot analysis of PARP cleavage in cell lysates of vehicle, sorafenib and lenvatinib treated PLKO and shPLXNC1 clones of PLC/PRF/5 cells (C). Western blot analysis is conducted on cell lysates from vehicle-, sorafenib-, and lenvatinib-treated PLKO and shPLXNC1 clones of Hep3B cells to assess PARP cleavage (D). GAPDH is used as a loading control. The relative levels of cleaved PARP are normalized to GAPDH and represented in the bar graph. The statistical analyses are conducted using Student’s t-test on the GraphPad Prism 8 software (*p < 0.05; **p < 0.01; ***p < 0.001).

**Figure 3:**
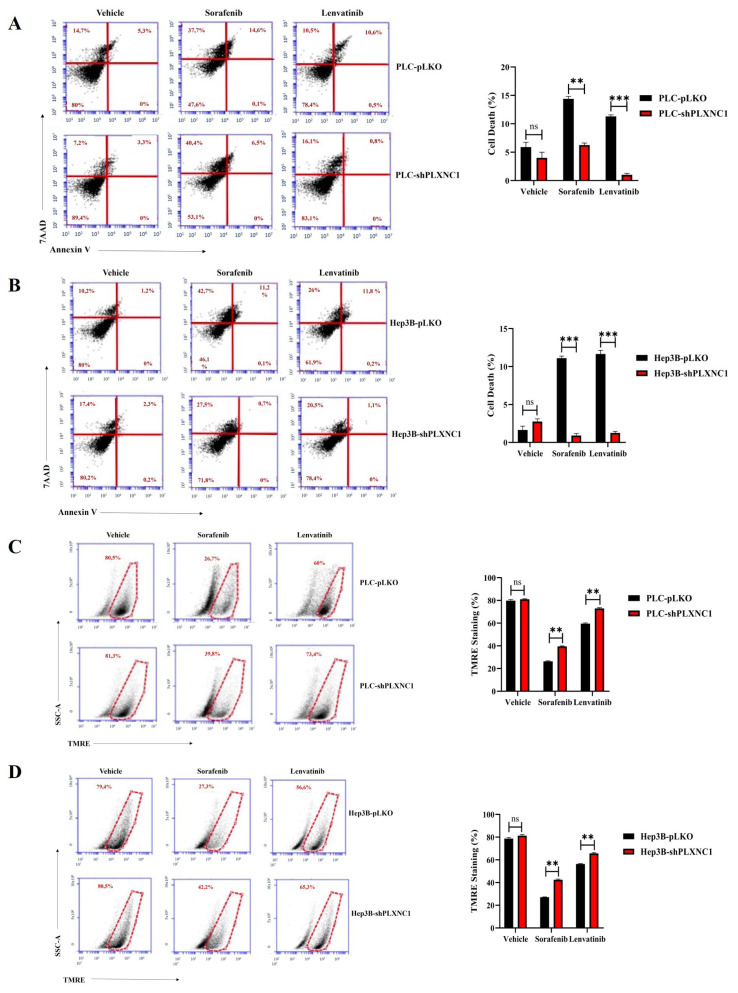
Flow cytometric analysis of cell viability and apoptosis of drug-treated cells. Cell death analyses of cells subjected to vehicle, sorafenib and lenvatinib treatments are conducted by Annexin V/7AAD staining and shown in flow cytometry scatter plots and bar charts for PLC (A) and Hep3B (B). The TMRE staining of the cells are analyzed by flow cytometry and the results are represented by scatter plots and a bar graph for PLC (C) and Hep3B cell clones (D). The statistical analyses are conducted using Student’s t-test on the GraphPad Prism 8 software (*p < 0.05; **p < 0.01; ***p < 0.001).

Given the resistance of EMT undergoing cells to drug therapy, we next investigated the effects of multi-kinase inhibitor treatment on shPLXNC1 knockdown and control clones of HCC cells. Sorafenib and lenvatinib were approved as first-line treatment for unresectable HCC cases (Leowattana et al., 2023). The first step involved treating pLKO and shPLXNC1 clones of PLC/PRF/5 and Hep3B cells with escalating doses of sorafenib and lenvatinib, with the aim of determining the IC50 values of the drugs through cell viability assay. These doses were then applied for the subsequent in vitro assays (Figures 2A–2B). Next, shPLXNC1 and control PLKO clones of PLC and Hep3B cells were administered with the IC50 doses of the drugs, and apoptotic death of cells was evaluated through the assessment of PARP cleavage on Western blot. There were no significant changes in PARP cleavage between cells treated with vehicle reagents. The shPLXNC1 cells of both PLC and Hep3B cells exhibited notable resistance to sorafenib and lenvatinib treatment in comparison to the pLKO clones (Figures 2C–2D). The results of the flow cytometric analyses of apoptotic cell death in drug-treated cells were found to be in accordance with those of the cleaved PARP assay. No significant changes were observed between the vehicle groups. The shPLXNC1 clones of both PLC and Hep3B cells demonstrated a marked and significant survival under drug treatment (Figures 3A–3B). The responses of cells to drug treatment were also monitored through the mitochondrial uptake of TMRE dye in living cells. No significant difference was observed between vehicle-administered cell clones of PLC and Hep3B. However, in conditions where both kinase inhibitors were applied, TMRE staining levels were significantly higher in shPLXNC1 clones of PLC and Hep3B cells compared to control clones of both cell lines (Figures 3C–3D). These results were consistent with reduced apoptosis and increased survival of drug-treated shPLXNC1 HCC cell clones, as detected by cleaved PARP and flow cytometry assays.

### 3.3. PLXNC1 expression and proliferation characteristics of PLXNC1 clones of PLC cells are preserved in tumor xenografts in nude mice

**Figure 4:**
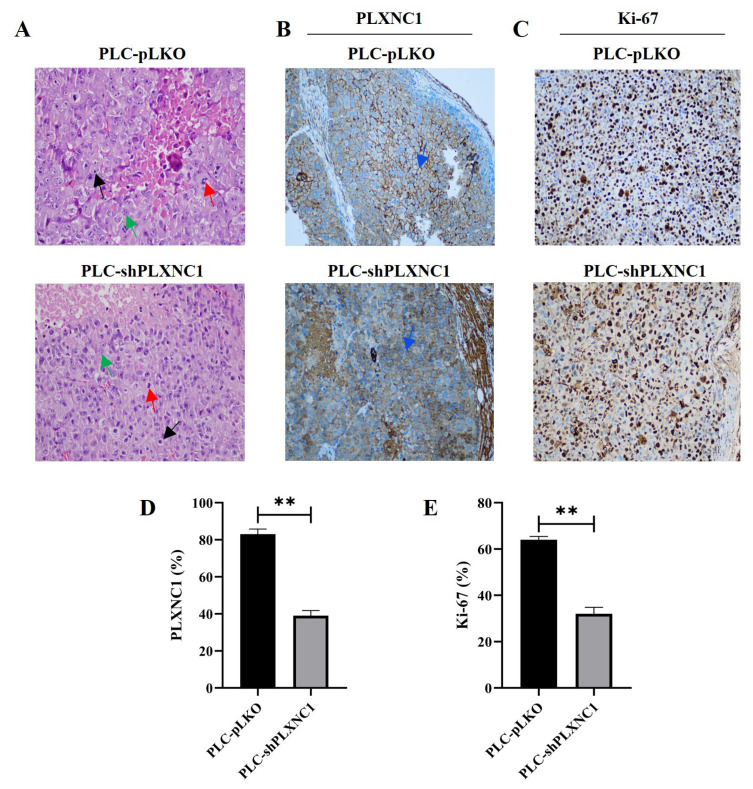
H&E and IHC staining of tumors established by pLKO and shPLXNC1 cell clones of PLC/PRF/5 cell line. Representative images of H&E staining (A) and IHC staining with Plexin C1 (B) and Ki-67 (C) antibodies of tissue sections from pLKO and shPLXNC1 tumors are shown. Tumor cells with large nuclei (black arrow), apparent nucleoli (green arrow) and high mitotic activities (red arrow) are shown in A. The blue arrows indicate the membrane expression of Plexin C1 in B. The mean Plexin C1 and Ki-67 staining values of pLKO and shPLXNC1 tumor sections are presented in (D) and (E), respectively (**p < 0.01).

PLC cells were selected for in vivo studies, and subcutaneous engraftment was performed on female BALB/c nude mice with both shPLXNC1 and pLKO control clones of this cell line. We started the drug administration when the tumor sizes reached 200 mm^3^ and the experiment was terminated after 28 days. Tumors were excised from the sacrificed mice and before proceeding with the assessment of the responses to drugs between experimental groups, we first adjusted the tumor engraftment by hematoxylin and eosin (H&E) staining in the untreated mice injected with shPLXNC1 and PLKO cell clones. Tumor cells with large nuclei, apparent nucleoli and high mitotic activities consistent with HCC characteristics were observed in both groups (Figure 4A). Then, the expression of PLXNC1 in these mice was investigated through immunohistochemistry. As anticipated, a diminished membrane staining was discerned in shPLXNC1 cells (Figure 4B). The proliferation rate between shPLXNC1 and control xenografts was evaluated by Ki-67 staining (Figure 4C). The mean membrane staining was 81% and 37% in the pLKO and shPLXNC1 groups, respectively, indicative of reduced PLXNC1 expression in PLXNC1-knockdown cells (Figure 4D). The mean Ki-67 staining values were 65% and 30% in the PLKO and shPLXNC1 groups, respectively, which is consistent with the observed inhibition of proliferation in vitro (Figure 4E).

### 3.4. PLXNC1 knockdown confers resistance to sorafenib and lenvatinib treatment in mouse tumor models

**Figure 5:**
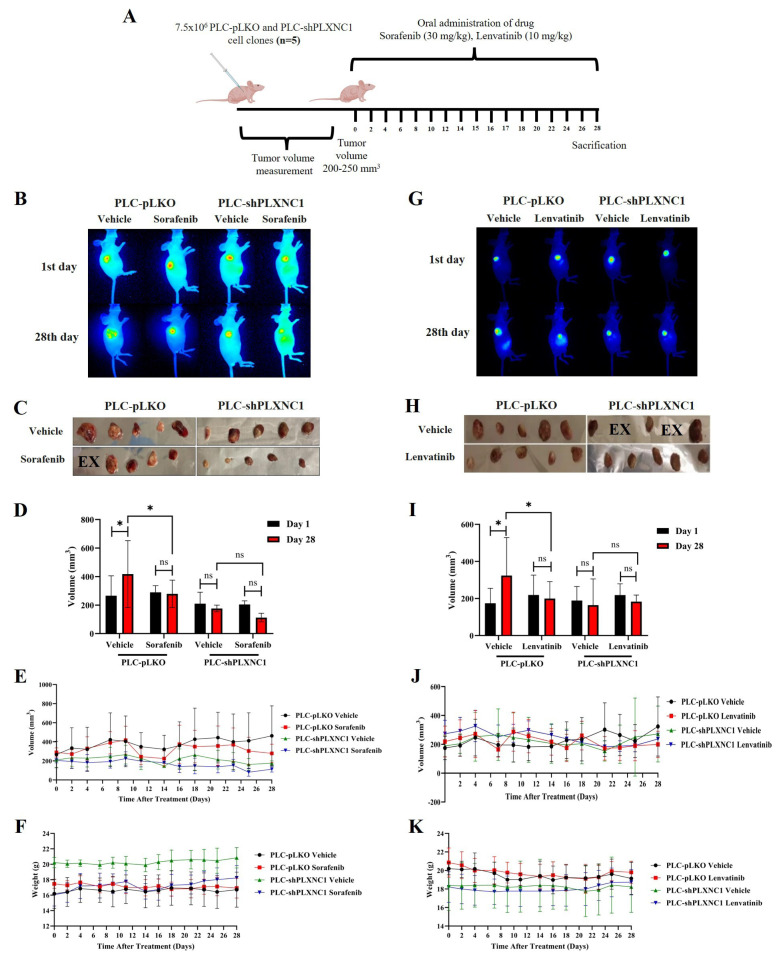
The effects of MKI treatment on mouse tumor models. The experimental process is schematized (A). Images show the variation in tumor size of sorafenib-treated (B) and Lenvatinib-treated (G) pLKO and shPLXNC1 mouse models. Photographs of excised tumors on day 28 for the sorafenib (C) and lenvatinib (H) groups. The variation in tumor volumes in response to sorafenib (D) and lenvatinib (I) on day 28. The evolution of tumor size over the course of the experiment is illustrated in (E) and (J), for the sorafenib and lenvatinib, respectively. The time-dependent weight change in mice under treatment with sorafenib (F) and lenvatinib (K) is illustrated. Student’s t-test was performed in GraphPad Prism 8 program (*p < 0.05; ***p < 0.001; ns: not-significant).

Eight therapy groups of 5 mice each were included in the study, and 20 mice were implanted with control pLKO and the other half with shPLXNC1 clones of PLC/PRF/5 cells. Groups are represented in the Table and, sorafenib, lenvatinib and their corresponding vehicle reagents were administered by oral gavage every three days (Figure 5A). The DMSO vehicle reagent of sorafenib treatment group did not show any therapeutic effect in the PLC-pLKO group of mice as the average tumor volume increased significantly at the endpoint of the experiment. However, tumor volumes remained stable when mice with PLC-pLKO tumors were treated with sorafenib, suggesting that the drug induced a growth arrest in HCC cells expressing PLXNC1. In the shPLXNC1 group, no significant difference was observed between the initial and final tumor volumes in mice that received either the vehicle or sorafenib. However, a more pronounced reduction in tumor volumes was evident in the sorafenib group (Figures 5B–5E). The results demonstrated that sorafenib administration impeded tumor growth in the pLKO group, indicating that this inhibitor is an efficacious therapeutic agent in PLXNC1-expressing hepatocellular carcinoma cells. A comparison of the PLC-pLKO and PLC-shPLXNC1 vehicle groups revealed a significant increase in tumor volume in the former on day 28, while the latter exhibited, albeit not significantly, a decrease in tumor volume. These findings suggest that the inhibitory effect of PLXNC1 silencing on cell proliferation may be responsible for these observations. The mice were monitored for weight loss over the course of the study, and no statistically significant differences were observed (Figure 5F). In the pLKO groups, the lenvatinib vehicle reagent PEG400 did not affect tumor progression. However, lenvatinib treatment resulted in tumor growth arrest, indicating that this inhibitor may have therapeutic potential for PLXNC1-expressing tumors. In contrast, no therapeutic effect on tumors was observed in mice implanted with shPLXNC1 cells and treated with lenvatinib. Nevertheless, tumor growth remained unaltered in both the vehicle- and lenvatinib-treated groups, indicating that the observed growth arrest was attributable to the antiproliferative impact of PLXNC1 silencing. This effect was more evident when the vehicle administered pLKO and shPLXNC1 groups were compared. Tumor growth was sustained in the presence of PLXNC1, whereas tumor growth was arrested when PLXNC1 was knocked down (Figures 5G–5J). Routine monitoring of the mice revealed no statistically significant weight change in the experimental groups over the course of the experiment (Figure 5K).

### 3.5. TUNEL assay demonstrates significant resistance of PLXNC1-silenced HCC cells to treatment with kinase inhibitors

**Figure 6:**
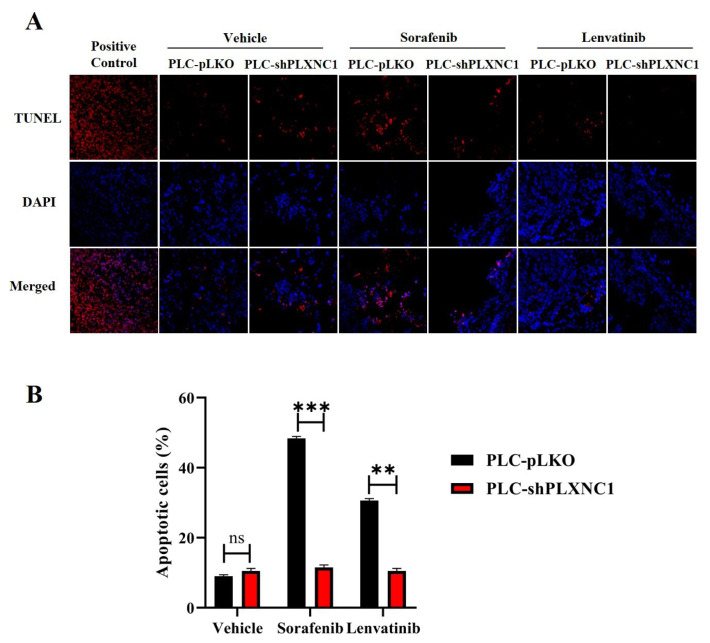
TUNEL detection of apoptotic cells in tumor tissue sections from treatment groups. (A) Representative fluorescence microscopy images of TUNEL stained cells. Red fluorescing cells are TUNEL positive apoptotic cells (first row). Cell nuclei are counterstained with DAPI (second row), and images are merged (third row). (B) Bar graph shows the apoptotic index values of vehicle-, sorafenib and lenvatinib-administered pLKO and shPLXNC1 tumor groups (**p < 0.01; ***p < 0.001).

In comparison to vehicle administration, both sorafenib and lenvatinib demonstrated the capacity to influence the growth of tumors in pLKO clones of PLC cells that express Plexin C1. However, no notable tumor response was discerned in shPLXNC1 clones (Figures 5D–5I). Therefore, to investigate the potential resistance of shPLXNC1 cells to pharmacological intervention, an in situ apoptosis TUNEL assay was conducted on tumor sections of pLKO and shPLXNC1 cells treated with vehicle, sorafenib and lenvatinib (Figure 6A). The administration of vehicles did not result in any notable differences in the mean apoptosis indexes of pLKO and shPLXNC1 clones (8.75 vs 10, respectively). However, the groups treated with sorafenib and lenvatinib reached mean AI values of 48.75 and 30.2, respectively, in the pLKO tumors. In stark contrast, the mean AI values were 11 for sorafenib and 10 for lenvatinib in shPLXNC1 tumors, indicating significant resistance to apoptosis of shPLXNC1 cells to inhibitor treatment (Figure 6B).

## 4. Discussion

Cancer-related deaths in patients with malignant tumors of epithelial origin occur because of metastatic disease. EMT is an embryonic program that regulates the migration of cells during development and is activated during cancer metastasis and tissue repair in adult life (Thiery and Sleeman, 2006). It is characterized by the repression of E-Cadherin that allows cancer cells to invade and metastasize (Kalluri and Weinberg, 2009). In HCC, multi-kinase inhibitors (MKIs) such as Sorafenib, Lenvatinib, Regorafenib, and Cabozantinib target various receptor tyrosine kinases to disrupt crucial signaling pathways involved in tumor growth and angiogenesis (Llovet et al., 2008; Xiang et al., 2014; Kudo et al., 2018; Zhang et al., 2021). Among these MKIs sorafenib and lenvatinib have been approved as first-line treatment of advanced unresectable HCC cases. Both sorafenib and lenvatinib target multiple signaling pathways including but not limited to Ras/Raf/MEK/ERK, c-kit, Flt3, VEGFR2/3, and PDGFR (Tohyama et al., 2014). However, lenvatinib also acts on FGFR signaling making it a more potent angiogenesis inhibitor than sorafenib (Matsuki et al., 2018), and lenvatinib-treated patients have better progression-free and overall survival than those treated with sorafenib (Hu et al., 2023). Although these MKIs have shown efficacy in improving survival outcomes, resistance often develops, necessitating second-line treatments with Regorafenib, and Cabozantinib (Shyam Sunder et al., 2023). The mechanisms of resistance to sorafenib and lenvatinib have been attributed to overexpression of EGFR, abnormal changes in the JAK/STAT pathway, activation of autophagy allowing HCC cells to survive nutrient deprivation, hypoxia within the tumor mass inducing VEGF through HIF-1α, dysregulated epigenetic regulation and EMT. Therefore, there is an urgent need to develop molecular markers to predict the sensitivity of HCC cells to sorafenib and lenvatinib. Among these factors, it is worth focusing on EMT in the context of this work, as we have previously shown that knocking out Plexin C1 induces EMT and its expression is inversely correlated with tumor differentiation, suggesting that it may contribute to the development of resistance to these MKIs. Our results clearly show that PLXNC1-expressing pLKO clones of HCC cells are sensitive to both sorafenib and lenvatinib treatment, as demonstrated in mouse tumor models where treatment with either drug halted tumor growth compared to vehicle treatment. However, tumors established with shPLXNC1 clones showed no difference in their response to vehicle and MKIs treatment. An interesting observation in the shPLXNC1 group was the decrease in tumor volume at the end of the experiment, which contradicts the results observed by others in gastric cancer (Chen et al., 2020). However, in this study, contrary to what we have seen, PLXNC1 expression was found to favor tumor growth and migration. Given the stationary state of shPLXNC1 tumors in both vehicle and drug treatments, we attributed the reduction in tumor size to the antiproliferative effect of PLXNC1 knockdown. Furthermore, the resistance of shPLXNC1 tumors to MKI treatment was evidenced by reduced cell apoptosis as measured by the TUNEL assay. Both slower proliferation rate and resistance to apoptosis are hallmarks of EMT, which we have shown to be induced by knockdown of PLXNC1 in HCC cells.

## 5. Conclusion

Plexin C1 expression makes HCC cells more sensitive to therapy with sorafenib and lenvatinib. However, its knockdown confers resistance to these therapies, yet tumors fail to grow due to inhibition of proliferation in the absence of PLXNC1. This dual aspect of PLXNC1’s effect on HCC cells adds another layer of complexity to the mechanistic understanding of the effects of multi-kinase inhibitors on HCC. Consequently, we propose that targeting PLXNC1 and its associated pathways could offer a novel approach to overcoming resistance and enhancing treatment efficacy in HCC patients.

## Figures and Tables

**Table:** The experimental groups of mouse tumor models.

Drug	PLC-pLKO cell clone (7.5 × 10^6^ cell/200 μL sc.)	PLC-shPLXNC1 cell clone (7.5 × 10^6^ cell/200 μL sc.)
**Vehicle (DMSO)**	5 BALB/c nude mice	5 BALB/c nude mice
**Sorafenib (30 mg/kg)**	5 BALB/c nude mice	5 BALB/c nude mice
**Vehicle (PEG-400)**	5 BALB/c nude mice	5 BALB/c nude mice
**Lenvatinib (10 mg/kg)**	5 BALB/c nude mice	5 BALB/c nude mice
